# Radiotherapy in langerhans cell histiocytosis - a rare indication in a rare disease

**DOI:** 10.1186/1748-717X-8-233

**Published:** 2013-10-09

**Authors:** Jan Kriz, Hans Theodor Eich, Frank Bruns, Reinhard Heyd, Ulrich Schäfer, Uwe Haverkamp, Jens Büntzel, Heinrich Seegenschmiedt, Oliver Micke

**Affiliations:** 1 Klinik und Poliklinik für Strahlentherapie, Universitätsklinikum Münster, Münster, Germany; 2 Klinik für Strahlentherapie und spezielle Onkologie, Medizinische Hochschule, Hannover, Germany; 3 Zentrum für Radiochirurgie und Präzisionsbestrahlung, Klinikum der J.W. Goethe Universität Frankfurt am Main, Frankfurt, Germany; 4 Klinik für Strahlentherapie, Klinikum Lemgo, Lippe-Lemgo, Germany; 5 HNO-Klinik, Südharz-Krankenhaus, Nordhausen, Germany; 6 Strahlenzentrum, Hamburg, Germany; 7 Klinik für Strahlentherapie und Radioonkologie, Franziskus Hospital, Bielefeld, Germany

**Keywords:** Langerhans cell histiocytosis, Radiotherapy, Benign disorder, Effectivity, Rare disease

## Abstract

**Introduction:**

Langerhans Cell Histiocytosis (LCH) represents a rare benign disorder, previously designated as “Histiocytosis X”, “Type II Histiocytosis” or “Langerhans Cell Granulomatosis”. Clinical presentation includes osteolysis, ulcerations of skin and soft tissues but also involvement of the CNS is described.

Because treatment concepts are not well defined the German Cooperative Group on Radiotherapy for Benign Diseases performed a retrospective analysis.

**Methods and material:**

Eight closely cooperating centres collected patients’ data of the past 45 years. As study endpoints disease free survival, recurrent disease, death and therapy related side effects were defined.

**Results:**

A total of 80 patients with histologically proven LCH were irradiated within the past 45 years. According to the LCH classification of Greenberger et al. 37 patients had stage Ia, 21 patients stage Ib, 13 patients stage II and 9 patients stage IIIb and the median age was 29 years. The median Follow up was 54 months (range 9–134 months). A total of 39 patients had a surgical intervention and 23 patients a chemotherapy regimen.

Radiation treatment was carried out with a median total dose of 15 Gy (range 3–50.4 Gy). The median single fraction was 2 Gy (range 1.8-3 Gy).

Overall, 77% patients achieved a complete remission and 12.5% achieved a partial remission. The long-term control rate reached 80%. Within an actuarial overall 5-year survival of 90% no radiogenic side and late effects ≥EORTC/RTOG II° were observed.

**Conclusion:**

In the present study a large collective of irradiated patients was analysed. Radiotherapy (RT) is a very effective and safe treatment option and even low RT doses show sufficient local control.

## Introduction

Langerhans cell histiocytosis (LCH) is a rare benign histiocytic disorder most commonly characterized by single or multiple osteolytic bone lesions but also ulcerations of skin and soft-tissues and also involvement of the CNS are described.

It is characterized by an uncontrolled clonal proliferation of Langerhans cells which belong to the normal human mononuclear-phagocytic system. The pathologic growth pattern remains still unclear but reactive and paraneoplastic processes have been discussed [1-4].

Previously designated as “Histiocytosis X”, “Hand-Schuller-Christian disease”, “Letterer-Siwe-Syndrom”, “Type II Histiocytosis” or “Langerhans Cell Granulomatosis” LCH involves single (single-system disease) or multiple (multiple-system disease) organ systems [5]. In 60% bony manifestations as single-system disease with uni- or multifocal lesions but also multi-system disease with activity in other organ systems are described [5]. In case of involvement of the CNS diabetes insipidus is a typical symptom [2]. Patients with single-system disease tend to do well. Patients with multiple-system disease sometimes have an unpredictable outcome but also in patients with only one bony lesion progression, disseminated disease and fatal outcome is described [6]. Nevertheless also self-healing and self-limiting courses are reported.

Radiotherapy (RT) is used since decades in the treatment of LCH and is effective even at low RT-doses. The first successful treatment was described in 1930 by Sosman [7]. Since then, the effectivity of radiotherapy in LCH has been shown in a large number of publications [4,8-12].

Choosing RT as a treatment option the following aspects should be respected: the age of patients, the possibility of radiogenic malignancies, and the semi-benign character of disease. It is used as a single treatment option for either bony lesions or other organ involvement but also in a multimodal approach in combination with chemotherapy (CTX), surgery or steroids [6,13-16]. The mechanism of actions induced by ionizing radiation target cells remains unclear [7,17-21]. Suppression of inflammatory processes induced by RT and RT-sensitivity of Langerhans cells are discussed [8,9,22].

In general, two radiotherapy indications must be distinguished: The treatment of painful or unstable uni- or multifocal bone lesions and the treatment of extra-osseous soft tissue or organ involvement [8].

Because treatment concepts, indication of RT, fractionation and timing of RT are not well defined this retrospective analysis was performed by the German Coopeartive Group for Benign Diseases (GCHBD) of the DEGRO.

## Methods and material

Eight German closely cooperating radiotherapy centres collected clinical features, treatment concepts and outcome data of their patients, treated for LCH during the past 45 years. Participating institutions were three academic, four non-academic and one private institution. To determine the efficacy of RT as study endpoints disease free survival, recurrent disease, death and therapy related side effects were defined.

## Results

Between 1966 and 2011 a total of 80 patients with histological proven LCH were irradiated. There were 45 women and 35 men with a median age of 29 years (range 9–81 years). According to the LCH classification of Greenberger et al. 37 patients had stage Ia, 21 patients stage Ib, 13 patients stage II and 9 patients stage IIIb (The staging system proposed by Greenberger et al. [14]). The median Follow up was 54 months (range 9–134 months). A total of 39 patients had a surgical intervention and 23 patients a chemotherapy regimen. 24 patients were irradiated “up-front” and in 43 patients RT was used as salvage therapy.

### The staging system proposed by Greenberger et al. [14]

Stage I 

a) Single monostotic bone lesion

b) Multiple lesions in one or multiple bone

Stage II >24 months of age at diagnosis and having one or more of the following systems involved: diabetes insipidus, teeth, gingivae, lymph nodes, skin, mild lung involvement (i.e., infiltrates seen on chest radiograph without pulmonary symptoms or gross consolidation), focally positive bone marrow

Stage III 

a) Age <24 months at diagnosis with any of the systems involved in stage II

b) Age >24 months with involvement of liver and/or spleen, massive nodal involvement (nodes > 5 × 5 cm in several sites above or below diaphragm), honeycomb lung (major involvement in all areas with apparent fibrosis), bone marrow packed

Stage IV Spleen > 6 cm (palpable below costal margin) and fever >1 month with or without any or all of the above systems involved

Stage V “Special” monocytosis in peripheral blood > 20% of differential cell count, in addition to stage III or IV

Overall 48 patients were treated at more than one site (range 2–8). Non-bony involvement was treated in 28 patients. The sites were as following: skin (n=8), lymph nodes (n=7), gingiva (n=5), soft tissue (n=5) and central nervous system (n=3).For bony involvement the treated sites were as following (more than one is possible): skull (n=14), rips (n=12), pelvis (n=8), humerus (n=5), clavicula (n=6), radius (n=3), ulna (n=3), femur (n=4), spine (n=8), hand (n=4), foot (n=3) and jaw (n=4).

The median number of follow-up visits was 3.5 times. Most patients received routiniously radiological examination.

Radiation treatment was carried out with a median total dose of 15 Gy (range 3–50.4 Gy). The median single fraction was 2 Gy (range 1.8-3 Gy). A total dose of 10 Gy or less was defined to be a low dose (n=26). Local control was not significant different in this low dose group compared with group of higher doses. Therefore a real dose response relationship could not be established.

**Figure 1:**
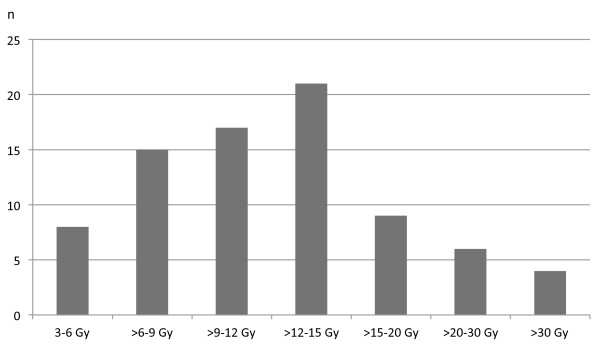
RT Doses.

Patients were treated with 60 Co gamma rays or 5-15MV photons of a linear accelerator (Figure 1).

The RT treatment planning was generally realized simulator based. In the past few years 3D CT-based and if available MRI based planning was preferred. The target volume definition included a safety margin ranging from 2 cm depending on the location of the tumor.

**Figure 2:**
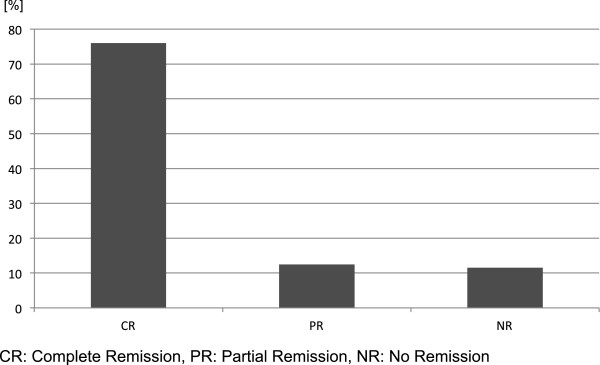
Results of RT [%] CR: Complete Remission, PR: Partial Remission, NR: No Remission.

Overall, 77% patients achieved a complete remission and 12.5% achieved a partial remission (Figure 2). The long term control rate reached 80%. The actuarial overall 5 year survival calculated by Kaplan-Meier Method was 90%.

Radiogenic side and late effects ≥EORTC/RTOG II° were not observed. No treatment related deaths and no secondary malignancies were observed and for 69 patients a positive documentation was available.

## Discussion

This analysis comprises a large collective of patients treated with radiotherapy for LCH. The following results emerge from this study:

–RT is a safe and effective treatment option for patients suffering from LCH.

–Even low RT doses show sufficient local control.

–Side effects are low and no treatment related deaths were observed.

**Figure 3:**
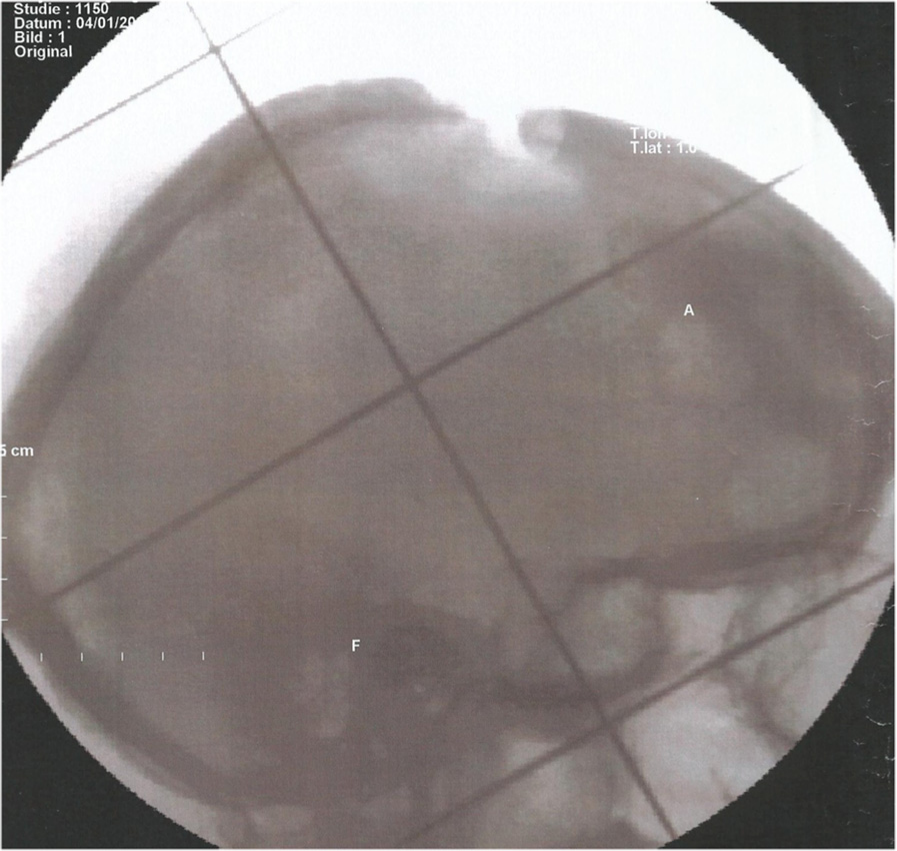
Radiation portal of a patient with massive osteolysis of the skull bone by LCH’.

**Figure 4:**
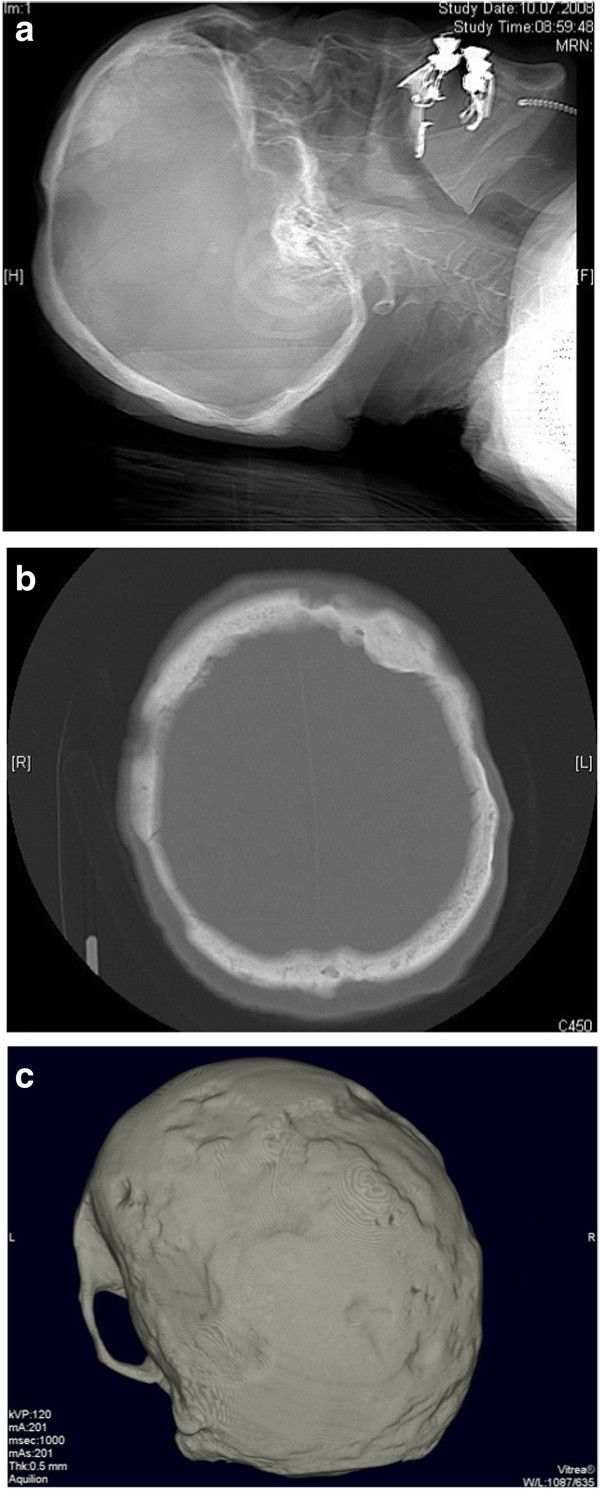
Pronounced recalcification of the skull bone four months after radiation treatment with a total dose of 10 Gy in: a) lateral radiograph b) transversal CT scan (bone window) c) 3D-CT reconstruction.

RT is used since decades in the treatment of LCH especially bony LCH and is even effective in low RT doses (Figures 3 and 4a/b/c). Due to the rarity of this disease treatment concepts are irregular and even the number of published cases in the literature are low. All studies reported in literature have a retrospective nature. Radiotherapy indications should respect the age of patients, the possibility of radiogenic malignancies, and the semi-benign character of disease.

In childhood LCH clear treatment recommendations and guidelines, e.g. by the Euro Histio Net, exist. But radiotherapy is generally not recommended due to the long term sequelae. In adult LCH there is a lack of such recommendations, but they are in preparation.

Therefore we conducted this study, to improve the knowledge on the effects of radiotherapy.

The largest studies in adult patients come from Klipatrick [6] and Greenberger [22].

Generally, most often patients are irradiated because of bony involvement, but also RT for patients with diabetes insipidus due to involvement of the skull base is described in the literature [6,20,21]. In a large analysis conducted by Klipatrick et al. the data of 263 patients with biopsy proven LCH were analysed. 40 patients suffered from diabetes insipidus. The role of RT in the treatment of diabetes insipidus is discussed controversial because low RT doses did not reverse the need for vasopressin therapy [6]. Although the authors present a large collective of patients with LCH, the fact that no information of RT doses and/or RT techniques are given must be critically considered. The analysis collected data from a single institution during 1915 to 1995, so probably old RT techniques were used that cannot be compared to today’s status. Greenberger et al. conducted a single centre study of 127 patients with LCH treated in a multimodal approach consisting of surgery, RT and chemotherapy. 21 of their patients were irradiated for diabetes insipidus and four of them reached complete reversal of symptoms [22]. A total 98 patients were irradiated because of bony lesions using RT doses ranging from 100 and 2000 rad. Local control was achieved in 95%.

Because treatment concepts are irregular due to the rarity of disease and most radiotherapeutic centres have low experience, Olschewski et al. conducted a patterns of care study. Only 23 of 123 (18.7%) participating institutions had experience in the treatment of LCH [21]. Most of the patients were treated because of bony involvement and doses ranged from 2 to 40 Gy. Complete remission was achieved in 77.5% of cases. These data are well comparable to our study, but it must be taken into account that some patients data analysed in the current study were also included in the patterns of care study.

Another study by Olschewski et al. studied the current literature. 142 studies were analysed. A Complete remission rate of 93% in patients with “single-system disease” and 76% in patients with “multi-system disease” was reported [21]. Also these findings are comparable to the outcome data of our study.

Another analysis that underlines the effectiveness of RT in the treatment of LCH is presented by El-Sayed et al. They showed the data of 17 patients. 15 of them were irradiated using doses from 30–35 Gy. Two of their patients suffered from diabetes insipidus and responded immediately to RT [20]. Most literature data concerning radiotherapy in adult LCH deal with in uni- or multifocal osseous single-system disease [16,19]. Summarizing the results in these bone lesions, the local control rates ranged from 75-100% and complete remission rates were up to 85%.

In our analysis data of 80 patients treated with RT are presented. Most of them (58%) suffered from painful bony involvement, but also patients with soft tissue affections, ulceration of the skin and CNS-involvement were treated. A total of 77% patients achieved a complete remission and 12.5% achieved a partial remission. The long term control rate reached 80%. The actuarial overall 5 year survival was 90%. These very good response data support the findings in the literature. Doses concepts range from 3 to 50.4 Gy. High doses were given in two patients that suffered from painful bony involvement of the femur. These findings can be also compared to the studies mentioned above [6,20,22].

The dose recommendation for radiotherapy is irregular and an exact dose-effect relationship has not been established yet. Doses applied are ranging from 1.4 Gy up to 45 Gy. In the treatment of adults doses from 10 to 20 Gy are recommended [8].

Total doses should be delivered in fractions of 1–2 Gy per day to avoid a possibly limited capacity for tissue repair mechanisms in larger single doses [8].

Side effects of radiotherapy are extremely rare due to the limited doses applied and depend on the irradiated site. Most literature data do not provide any information about acute side effects of radiotherapy [8] and also in our analysis no side effects > EORTC/RTOG II° and no treatment related deaths were observed.

Late side effects of radiotherapy are also rare due to the low applied dose range [8,19]. The only concern, that should be considered, is the extremely low danger of radiation-induced risk of second malignancies. Greenberger et al. [22] reported a rate of 3.9% for induction of malignant tumors. This analysis has to be seen critically due to the fact that many children were treated in this cohort [8,23,24].

Surely, there is some concern about using RT in a non-malignant disease especially as patients are often infants. The risk of malignant change in the irradiated area many years later should be taken seriously. Nowadays in the megavoltage era the risk is not as high as it was in the orthovoltage era where many patients whose data are now published, were treated. In addition also the risk of the other treatment options especially chemotherapy should be considered.

There are several publications that described second malignancies and induction as e.g. leukemias, teratoma, malignant menigeomas and osteosarcoms [8,19]. The fact, that also children were treated should be seen critically because they have a larger risk of cancer induction and there is a well-known tendency of patients with LCH to develop malignancies independent of therapy.

Finally, the radiation treatment techniques have changed and improved substantially during the last decades. Nevertheless, the indication for radiotherapy for a palliative treatment in LCH has to be chosen carefully and possible risks must be taken into account [8].

## Conclusion

The present analysis comprises a large collective of patients treated with histologically proven LCH. Our study is multicentric in contrast to the most studies were only single institutional data were published. The outcome data of our study underline the effectiveness of RT in the treatment of LCH. RT is a safe and simple treatment option. Even low RT doses show sufficient local control and side effects are generally low.

Possible indications for a radiation treatment are:

–Unresectable lesions if a resection would significantly compromise anatomic function.

–Recurrent or progressive lesions.

–Adjuvant treatment following marginal or incomplete resection.

–Painful or otherwise symptomatic lesions compromising quality of life.

## Competing interests

The authors declare that they have no competing interests.

## Authors’ contributions

All authors treated patients in this study. JK drafted the manuscript. All authors read and approved the final manuscript.
